# Supplementary material for: Laguncularia racemosa Phenolics Profiling by Three-Phase Solvent System Step-Gradient Using High-Performance Countercurrent Chromatography with Off-Line Electrospray Mass-Spectrometry Detection
Source: Molecules. 2021 Apr 15;26(8):2284. doi: 10.3390/molecules26082284 (PMC8071337; doi:10.3390/molecules26082284)
Supplement: Supplementary file 1 [file molecules-26-02284-s001.zip › molecules-1175296-supplementary.pdf]

Laguncularia racemosa **metabolite profiling by three-phase solvent system step-gradient using high-performance countercurrent chromatography with *off-line* ESI-MS/MS detection**

Fernanda das Neves Costa [1*], Gerold Jerz [2], Peter Hewitson [3],

Fabiana de Souza Figueiredo [1], Svetlana Ignatova [3]

[1] Federal University of Rio de Janeiro, Institute of Natural Products Research, CCS, Bloco H, Ilha do Fundão, RJ 21941-11 590, Brazil

[2] Institute of Food Chemistry, Technische Universität Braunschweig, Schleinitzstrasse 20, 38106 Braunschweig, Germany

[3] Advanced Bioprocessing Centre, Department of Chemical Engineering, Brunel University London, UB8 3PH, UK

*fncosta@ippn.ufrj.br

Supplementary Material

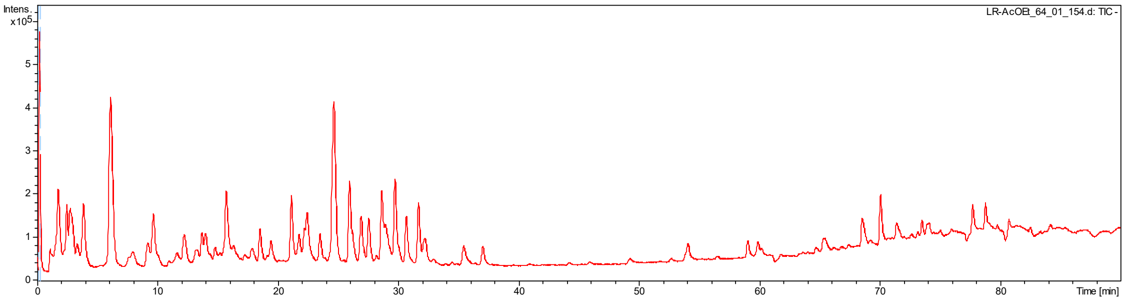

Supplementary Figure S1. EtOAcPart preliminary analysis by LC-ESI-TOF-MS. The mobile phase was methanol (B) and water (A) containing 0.05% (v/v) formic acid. The linear gradient elution was set from 10% to 100% of B in 90 min.

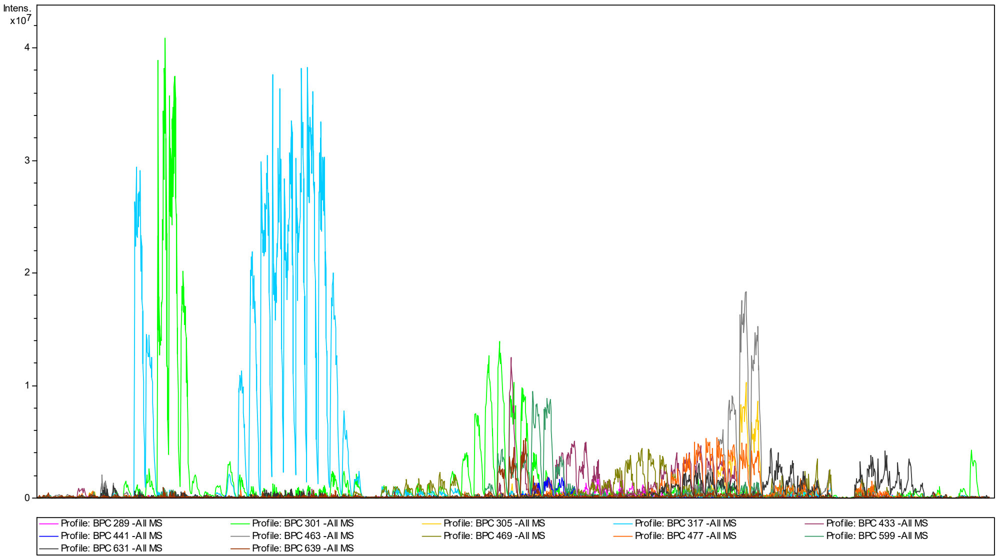

(a)

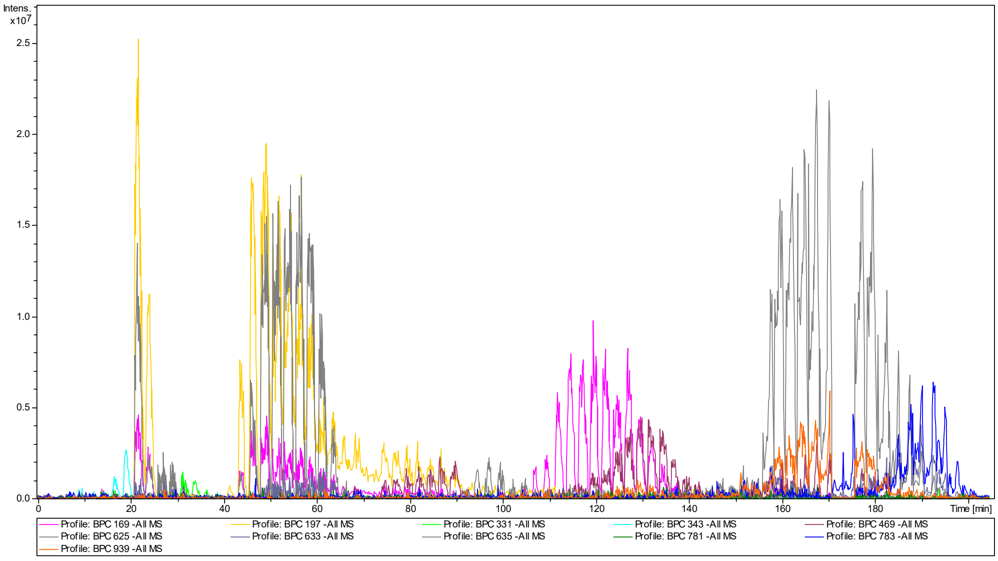

(b)

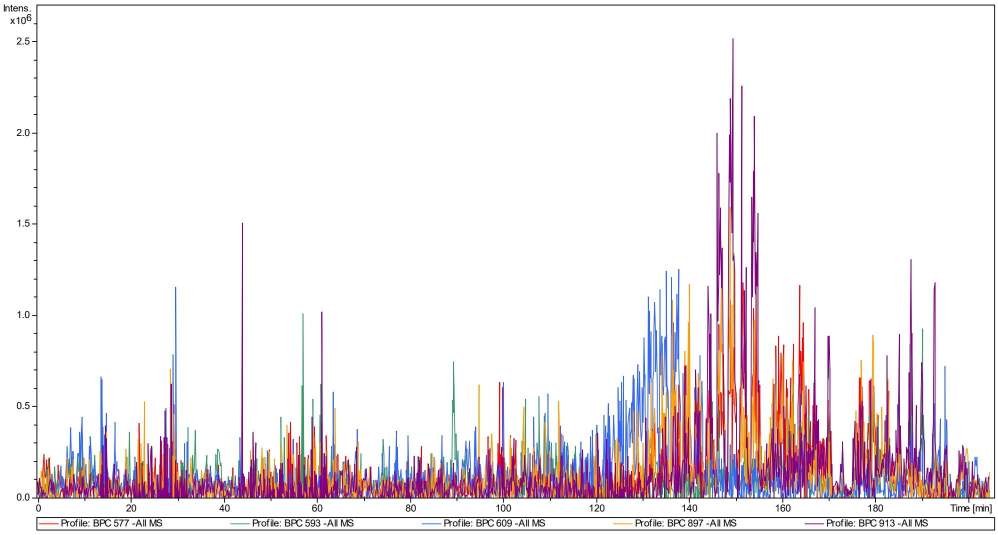

(c)

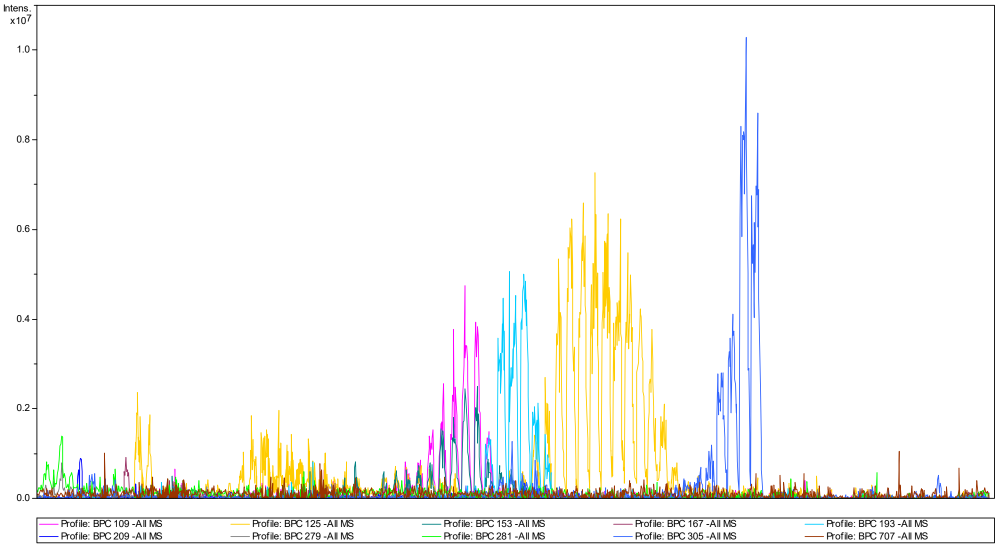

(d)

Supplementary Figure S2. HPCCC off-line injection ESI-MS/MS profile of the the EtOAc Part by use of selected single ion traces for target compounds or classes. (a) Flavonoids, (b) Hydrolysable tannins, (c) Condensed tannins and (d) other compounds.

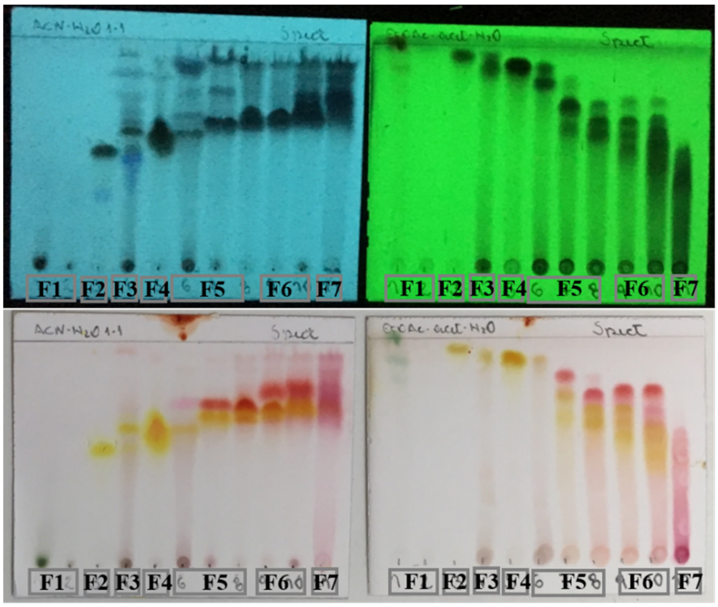

Supplementary Figure S3. TLC analysis of *L. racemosa* CCC combined fractions. On the left: reversed phase silica gel TLC plates developed with acetonitrile-H_2O 1:1 (v/v). On the right: Normal phase silica gel TLC plates developed with EtOAc-acetone-H_2O 25:15:10 (v/v/v). Visualization was done using λ 254 nm UV light and spray-reagent H_2SO_4 10% and vanillin 5%. F means a group of jointed fractions according to TLC similarity.

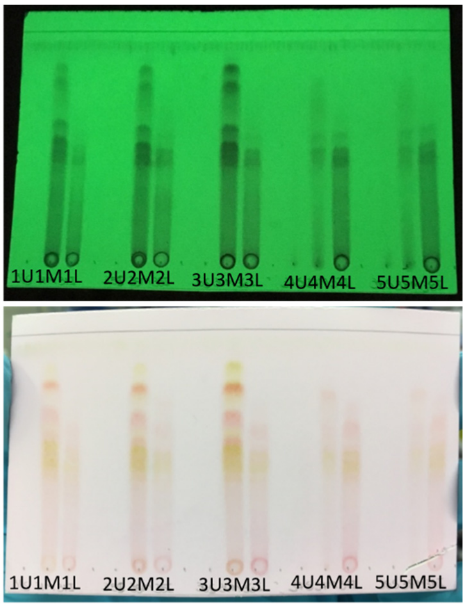

Supplementary Figure S4. Three-phase solvent system test by TLC. HPCCC solvent system: *n*-hexane-*tert.*butyl-methyl ether-ACN-H_2O **(1)** 1-1-2-1, **(2)** 2-1-3-2, **(3)** 2-2-3-2, **(4)** 2-3-3-2 and **(5)** 3-5-5-2 (v/v); **(U)** upper, **(M)** middle and **(L)** lower phases. Normal

phase silica gel TLC plates developed with EtOAc-acetone-H_2O 25:15:10 (v/v/v), visualized using λ 254 nm UV light and spray-reagent H_2SO_4 10% and vanillin 5%.
